# 

**Published:** 1889-08-17

**Authors:** 


					r wtme V \VI ISi Per Annum. 6s. Kd.. nost iw
m. 151, Vol. VI-
-August 17th, 188S.
/?? Monthly Parts, 6d.; post free 7|d.
Registered at the General Post Office as a Newspaper.] ^ ^ Pcl' -tlnnuin> 's- oa?j POSl iree

				

